# Incorporating phylogenetic-based covarying mutations into RNAalifold for RNA consensus structure prediction

**DOI:** 10.1186/1471-2105-14-142

**Published:** 2013-04-27

**Authors:** Ping Ge, Shaojie Zhang

**Affiliations:** 1 Department of Electrical Engineering and Computer Science, University of Central Florida, Orlando, FL 32816-2362, USA

## Abstract

**Background:**

RNAalifold, a popular computational method for RNA consensus structure prediction, incorporates covarying mutations into a thermodynamic model to fold the aligned RNA sequences. When quantifying covariance, it evaluates conserved signals of two aligned columns with base-pairing rules. This scoring scheme performs better than some other approaches, such as mutual information. However it ignores the phylogenetic history of the aligned sequences, which is an important criterion to evaluate the level of sequence covariance.

**Results:**

In this article, in order to improve the accuracy of consensus structure folding, we propose a novel approach named PhyloRNAalifold. It incorporates the number of covarying mutations on the phylogenetic tree of the aligned sequences into the covariance scoring of RNAalifold. The benchmarking results show that the new scoring scheme of PhyloRNAalifold can improve the consensus structure detection of RNAalifold.

**Conclusion:**

Incorporating additional phylogenetic information of aligned sequences into the covariance scoring of RNAalifold can improve its performance of consensus structures folding. This improvement is correlated with alignment characteristics, such as pair-wise identity and the number of sequences in the alignment.

## Background

The discovery of novel non-coding RNA (ncRNA) families expanded our understanding of RNAs, which not only carry genetic codes for protein synthesis but also participate in other functions, especially the regulatory processes, such as localization, replication, translation and degradation [1-4]. In mammals, a substantial amount of transcripts (above 90%) are non-protein-coding, and most of them are functional [5,6]. What’s more, the non-coding regions in the human genome are crucially important. For example, microRNA (miRNA) is used as a marker to differ normal tissues from tumors [7-9]; long non-coding RNA (lncRNA) also contributes to human disease etiology [10]. These findings fuel the research of RNA and also pose new challenges.

Unlike protein-coding genes, whose primary sequences can be applied for accurate functional prediction with statistical signals, RNAs’ functions depend on their secondary structures. Many computational methods have been proposed to fold RNA structures. One type of popular algorithms adopts Minimum Free Energy (MFE) model to fold a single RNA sequence, which has been implemented in Mfold [11] and RNAfold [12]. However, the structure prediction accuracy of this approach is limited. One major reason is that the precise energy parameters are hard to obtain experimentally [13]; on the other hand, the functional RNA structure may not be the one with the minimum energy [4]. What’s more, single sequence folding may not be applied to discover new RNA families even if the predicted structures are correct, because the statistical signals in an RNA secondary structure are not strong enough to distinguish itself from the stable structures folding from random sequences [14,15].

Comparative methods can solve these problems by folding a consensus structure from multiple sequences, which not only improve the structure prediction accuracy, but also provide additional signals to discover novel RNAs [16]. The idea of this approach is that RNA secondary structures are conserved through evolution. Therefore, a consensus structure detected by comparing related RNA sequences should be more accurate and significant than the structure folded from a single sequence. With a consistent consensus structure, the specific structure of each sequence in the alignment can be obtained by constraint folding. A classic comparative method is the Sankoff algorithm [17]. Because constructing a precise structural alignment of RNA sequences is also a challenging problem, the Sankoff algorithm computes alignment and fold structure simultaneously. Excessive computational resources (*O*(*n*^6^)) are required by the Sankoff algorithm for a large-scale problem. Some implementations of this approach, such as Dynalign [18], Foldalign [19], LocARNA [20] and Conan [21], attempt to restrict its solution space by limiting the number of possible sub-structures. However these methods are still computationally expensive (*O*(*n*^4^)).

To reduce the computation complexity, comparative methods may align related sequences first and then detect conserved signals in the alignment to infer a consensus structure. One type of these methods extends the energy-based model from single sequences to alignments. Based on the assumption that high covariance of two aligned columns implies the conservation of pairing, all potential pairing columns in an alignment can be determined. After that the optimal consensus structure with minimum average free energy can be folded just as a single sequence structure. An example of covariance scoring scheme is Mutual Information (MI), which can measure the dependence of two columns in the alignments [22-24]. RNAalifold [25] adopts the basic idea of MI scoring and imports the pairing rules of RNA into the measurement of covariance. Another type of comparative methods is evolution-based. In these methods, no thermodynamic parameters but statistical learning algorithms are used. The evolutionary history of the aligned sequences is reformed with probability theories [26,27], and the RNA secondary structures are modeled as stochastic context-free grammar (SCFG) [28-30]. Both strategies have their own strengths and weaknesses [31]. Some methods try to integrate both approaches. For example, PETfold extends Pfold [30], an evolution-based algorithm, to consider the energetically favorable base-pairs [32]. However, PETfold utilizes a Nussinov style model [33], which does not make full use of the energy parameters. RNAalifold also tested two other covariance scoring schemes to incorporate evolutionary information [25,34], but neither of them yielded a better result.

In this article, we propose a novel method called PhyloRNAalifold. It improves RNAalifold by explicitly incorporating the phylogenetic tree of the aligned sequences into the computation of covariance scores. Like RNAalifold, PhyloRNAalifold detects pairing columns by evaluating covarying mutations and folds RNA structures through an MFE model. Unlike RNAalifold, which does not consider the relative positions of sequences in the phylogeny, PhyloRNAalifold counts the number of covarying mutations on the phylogenetic tree for each pair of columns with a parsimony approach. What’s more, comparing with PETfold, PhyloRNAalifold retains the Turner’s model [35] in RNAalifold, which describes RNA structures with many thermodynamic parameters derived from physical studies. With the supports of both energy-based model and evolution-based model, PhyloRNAalifold may detect consensus structures more precisely.

The rest of the article is organized as follows: in the methods section, we discuss the basic mechanism of RNAalifold, its shortcomings, and details of the PhyloRNAalifold algorithm. In the results section, we describe the benchmark datasets, experimental results, and the effect of parameters and alignment characteristics on our algorithm. In the discussion and conclusion section, we summarize our existing works and propose directions for future research.

## Methods

### Consensus folding energy and covariance score in RNAalifold

The basic approach of RNAalifold [25] is to integrate covarying mutation into the thermodynamic model to predict consensus structures. First, covariance scores are computed for all pairs of columns to determine possible pairing positions in the consensus structure. Then, based on the MFE model, the minimum average folding energy is computed with dynamic programming. Assume the given alignment is denoted by  , which contains *N* sequences $A=\{s^{1},s^{2},\ldots ,s^{N}\}$. Each sequence contains *L* symbols, including nucleotides and gaps, and $s_{i}^{k}$ represents the *i*^*th*^ symbol (1≤*i*≤*L*) at the *k*^*th*^ (1≤*k*≤*N*) RNA sequence. The minimization of free energy is computed by using the following recursive functions:

(1) $$\begin{array}{l} \;F_{i,j}=\text{min}(F_{i+1,j},\underset{i<k\leq j}{\text{min}}(C_{i,k}+F_{k+1,j}))\\ \;C_{i,j}=\phi_{2}\gamma_{i,j}+\text{min}\;\left\{\begin{array}{l} \underset{s^{k}\in A}{\sum }H(i,j,s^{k})\\ \underset{i<p<q<j}{\text{min}}\left(\underset{s^{k}\in A}{\sum }I(i,j,p,q,s^{k})+C_{p,q}\right)\\ \underset{i<p<j}{\text{min}}\left(FM_{i,p}+\text{FM}1_{p+1,j}+M_{a}\right) \end{array}\right)\;\\ \;FM_{i,j}=\text{min}\;\left\{\begin{array}{l} FM_{i+1,j}+M_{c}\\ \underset{i<p<j}{\text{min}}C_{i,p}+FM_{p+1,j}+M_{b}\\ \text{FM}1_{i,j} \end{array},\;\right)\\ \;\text{FM}1_{i,j}=\text{min}\;\left(\text{FM}1_{i,j-1}+M_{c},C_{i,k},\;\right) \end{array}$$

where *F*_*i*,*j*_,*C*_*i*,*j*_,*FM*_*i*,*j*_,*FM*1_*i*,*j*_ denote the minimum free energies for the region between *i*^*th*^ column and *j*^*th*^ column with unconstrained structure, with enclosed structure, with a multi-loop, and with a multi-loop containing a single branch, respectively. *H*(*i*,*j*,*s*) is the free energy for a hairpin loop enclosed by *s*_*i*_ and *s*_*j*_, and *I*(*i*,*j*,*p*,*q*,*s*) is the free energy for an internal loop containing two base-pairs, one is between *s*_*i*_ and *s*_*j*_ and the other is between *s*_*p*_ and *s*_*q*_. *M*_*a*_,*M*_*c*_ are penalties for closing bases and non-pairing bases in multi-loops. *M*_*b*_ is the bonus for branch bases in multi-loops.

The recursive functions were derived from the Turner’s model [35]. One major change made by RNAalifold for consensus folding is the usage of covariance score *γ*. It is not only a factor in the computing of free energy, but also determines the possible pairing columns in the alignment. Two parts, one is bonus and the other is penalty, are in this score. The first part of the covariance score is called the conservation score. For $\left(s_{i}^{k},s_{j}^{k}\right)$ and $\left(s_{i}^{l},s_{j}^{l}\right)$, three levels of confidence for pairing are assessed: base-pairs without mutation, base-pairs with one mutation, and base-pairs with two mutations. In the latest version of Vienna RNA package (v2.0) [36], the recursive function for computing conservation score is:

(2) $$\begin{array}{l} V_{i,j}\;=\;\frac{1}{N}\;\;\underset{1\leq k<l\leq N}{\sum }\;\;\left\{\begin{array}{ll} \;h(s_{i}^{k},s_{i}^{l})\;+h(s_{j}^{k},s_{j}^{l}) & \text{if}\;(s_{i}^{k},s_{j}^{k})\;\in \;B\;\text{and}\;(s_{i}^{l},s_{j}^{l})\;\in \;B\\ 0 & \text{otherwise} \end{array}\right) \end{array}$$

where *h*(*x*,*y*) is the Hamming distance between base *x* and base *y*, and  ={‘AU’, ‘UA’, ‘CG’, ‘GC’, ‘GU’, ‘UG’} is the set of all possible base-pairs. The second part is the penalty score *Q*_*i*,*j*_, which deals with a pair of symbols that cannot form a base-pair:

(3) $$Q_{i,j}=\underset{1\leq k\leq N}{\sum }\left\{\begin{array}{ll} 0 & \;\text{if}\;(s_{i}^{k},s_{j}^{k})\in B\\ 0.25 & \;\text{if}\;s_{i}^{k}\;\text{and}\;s_{j}^{k}\text{are gaps}\\ 1 & \;\text{otherwise} \end{array}\right)$$

Overall, the covariance score is:

(4) $$\gamma_{i,j}=V_{i,j}-\phi_{1}\times Q_{i,j}$$

where *ϕ*_1_=*ϕ*_2_=1. A threshold value *γ*_*t*_=−2 is defined for *γ*_*i*,*j*_. If *γ*_*i*,*j*_>*γ*_*t*_, *i*^*th*^ column and *j*^*th*^ column are considered to be pairing columns. In the final output, the minimum average folding energy, including the covariance score, is normalized by dividing *N*.

### Phylogenetic-based covarying mutation

**Figure 1:**
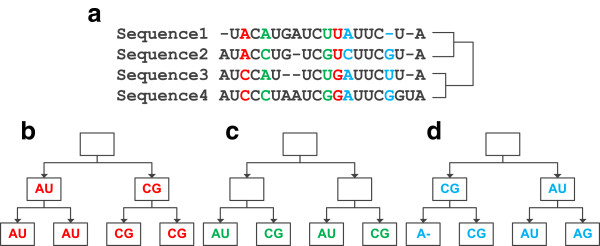
**Covarying mutations in an RNA alignment.** (**a**) A multiple RNA alignment and its phylogenetic tree. Three pairs of columns, which are marked with different colors, are analyzed in the following three sub-figures. (**b**) Possible covarying mutations in the red columns. In this case, only one pair-wise mutation is required at the root node. (**c**) Possible covarying mutations in the green columns. At least two pair-wise mutations occur at the internal nodes in this case; (**d**) Possible covarying mutations in the blue columns. There are non-pairing bases, ‘AG’ and ‘A-’. The label inference of the internal nodes does not depend on them. So in this case, the number of mutations is one.

RNAalifold incorporates covarying mutations into consensus folding to improve the detection of pairing columns. From Equation (2), it can be seen that RNAalifold counts the level of covariance by treating all sequences equally and try all possible combinations of base-pairs. In short, RNAalifold models the relationship of sequences as a complete graph. As a result, the specific evolutionary relationship among sequences in the phylogenetic history is ignored. Take the RNA structural alignment in Figure 1 as an example. The red and green columns achieve the same covariance score (2) in RNAalifold. However, as described in [37], the conservation evidence in Figure 1(c) is stronger than that in Figure 1(b) because at least two mutations occur at the green columns while only one is required to form the red ones.

PhyloRNAalifold models the relationship of aligned sequences as a tree by introducing the phylogenetic history of the alignment into the computation of covariance scores. The level of structural conservation is measured by the number of covarying mutations on the tree. Our assumption is that more covarying mutations on the tree mean stronger evidence of conservation. In addition, PhyloRNAalifold does not discard the original scoring scheme of RNAalifold, because experimental results showed this scheme can infer significant RNA structural aspects with high sensitivity and selectivity [38]. Assume *m*_*i*,*j*_ covarying mutations occur between *i*^*th*^ and *j*^*th*^ columns on the alignment ’s phylogenetic tree and the number of base-pairs on those columns is *b*_*i*,*j*_. The value of *m*_*i*,*j*_ depends on the size of the alignment. Since our approach focuses on improving the bonus part of the covariance scores, the number of covarying mutations is normalized with its upper bound: $\frac{m_{i,j}}{b_{i,j}-1}$. A new factor for the conservation score is proposed:

(5) $${∊}_{i,j}=1+\beta \times \frac{m_{i,j}}{b_{i,j}-1}$$

where *β* is the scale parameter for the normalized covarying mutation numbers. PhyloRNAalifold computes covariance scores with the following formula:

(6) $$\gamma_{i,j}^{p}={∊}_{i,j}\times V_{i,j}-\phi_{1}\times Q_{i,j}$$

All the other parameters and their default values in RNAalifold are retained. Due to the fact that $\gamma_{i,j}^{p}\geq \gamma_{i,j}$ (*∊*_*i*,*j*_≥1), two columns would be marked as pairing in PhyloRNAalifold if their covariance score in RNAalifold is greater than the threshold *γ*_*t*_ (the default value of *γ*_*t*_ is -2). Thus the advantage of PhyloRNAalifold is to import more potentially pairing positions with high mutation numbers.

### Computing the number of covarying mutations

Given a phylogenetic tree and labels at its leaves, the Fitch algorithm can optimize nucleotide assignment of the internal nodes to minimize the number of mutations [39]. If we model solving phylogeny as a maximum parsimony problem, this number can be taken as the actual number of mutations. The Fitch algorithm consists of a forward phase and a backward phase. In the forward phase, all possible labels at each internal node are inferred. In addition, the number of mutations is estimated during a bottom-up traversal. In the backward phase, a top-down pass is performed to find the optimal label at each internal node. Only the forward algorithm is applied to PhyloRNAalifold, since we do not need the exact labels at the internal nodes, but only the number of mutations on the tree. Without loss of generality, we require  to be a rooted binary tree. *r* denotes the root of  and *v*, *v*_*l*_, *v*_*r*_ denote a node, left child of *v*, and right child of *v* respectively. *F*(*v*) is the set of possible labels at node *v*, and *cost*(*v*) is the number of mutations on the sub-tree which is rooted at *v*. Then the forward phase can be described with the following recursive functions:

(7) $$\begin{array}{l} \begin{array}{l} \;F(v)=\left\{\begin{array}{ll} F(v_{l})\cap F(v_{r}) & \;\text{if}\;F(v_{l})\cap F(v_{r})\neq \emptyset \\ F(v_{l})\cup F(v_{r}) & \;\text{otherwise} \end{array}\right)\\ \;\text{cost}(v)=\left\{\begin{array}{ll} \text{cost}(v_{l})+\text{cost}(v_{r}) & \;\;\text{if}\;F(v_{l})\cap F(v_{r})\neq \emptyset \\ \text{cost}(v_{l})+\text{cost}(v_{r})+1 & \;\;\text{otherwise} \end{array}\right) \end{array} \end{array}$$

For each leaf, *F*(*v*) is a base at the corresponding sequence. After the computation is finished, *cost*(*r*) shows the minimum number of mutations on the phylogenetic tree. The optimization of this algorithm was proved in [40].

In Equation 5, the computation of *∊*_*i*,*j*_ does not depend on non-pairing bases. Therefore, in the revised Fitch algorithm non-pairing bases need not to be considered when the number of covarying mutations is computed. We changed the original Fitch algorithm in two ways: (1) at any leaf node, if $(s_{i}^{k},s_{j}^{k})\notin B$, set $(s_{i}^{k},s_{j}^{k})=$ (‘-’, ‘-’); (2) for one internal node *v*, if the bases at *v*_*l*_(*v*_*r*_) is (‘-’, ‘-’), *v* will obtain *F*(*v*_*r*_)(*F*(*v*_*l*_)) as its label. One example of this algorithm is shown in Figure 1(d). The revised Fitch algorithm can be described by using the following functions:

(8) $$\begin{array}{c} \begin{array}{l} \;F(v)=\left\{\begin{array}{ll} F(v_{l})\cap F(v_{r}) & \;\text{if}\;F(v_{l})\cap F(v_{r})\neq \emptyset \;\text{and}\;F(v_{l})\neq \text{(‘-’, ‘-’)}\;\text{and}\;F(v_{r})\neq \text{(‘-’, ‘-’)}\\ F(v_{l}) & \;\text{if}F\;(v_{r})=\text{(‘-’, ‘-’)}\\ F(v_{r}) & \;\text{if}\;F(v_{l})=\text{(‘-’, ‘-’)}\\ F(v_{l})\cup F(v_{r}) & \;\text{otherwise} \end{array}\right)\\ \text{cost}(v)=\left\{\begin{array}{ll} \text{cost}(v_{l})+\text{cost}(v_{r}) & \text{if}F(v_{l})\cap \;F(v_{r})\neq \emptyset \;\text{and}\;F(v_{l})\neq \text{(‘-’, ‘-’)}\;\text{and}\;F(v_{r})\neq \text{(‘-’, ‘-’)}\\ \text{cost}(v_{l}) & \text{if}F(v_{r})=\text{(‘-’, ‘-’)}\\ \text{cost}(v_{r}) & \text{if}\;F(v_{l})=\text{(‘-’, ‘-’)}\\ \text{cost}(v_{l})+\text{cost}(v_{r})+1 & \text{otherwise} \end{array}\right) \end{array} \end{array}$$

It is easy to see that our algorithm is optimal, because it only excludes non-pairing bases from the computation of the original Fitch algorithm.

In PhyloRNAalifold, the tree structure is an input variable and the clients can use any phylogenetic tree construction algorithm to build it. The time complexity of the original RNAalifold algorithm is *O*(*m*×*n*^2^+*n*^3^) [41], where *n* is the length of the alignment and *m* is the number of sequences in the alignment. The extra computation in PhyloRNAalifold is caused by the revised Fitch algorithm, whose time complexity ranges from *O*(log*m*) to *O*(*m*). In addition, PhyloRNAalifold needs to compute *∊*_*i*,*j*_ for each pair of columns in the alignment. Thus the overall time consumption of the revised Fitch algorithm is *O*(log*m*×*n*^2^) or *O*(*m*×*n*^2^). Neither of them increases the time complexity of RNAalifold.

## Results

### Benchmarking datasets

The 19 Rfam [42] families used in the CMfinder paper [43] were selected as our first benchmarking dataset. It captures the diversity of known families by excluding highly conserved ones. Other programs, such as PETfold [32] and RNAalifold [34], also adopted it in their experiments. All the testing families came from Rfam version 11.0 and their seed alignments were used. In order to evaluate the dependence of our folding algorithm on the alignment quality, we also realigned the seeds with ClustalW [44] to generate the second benchmarking dataset. For this dataset, the predicted structure of the first sequence in each alignment was compared with its real consensus structure to measure the accuracy. Pair-wise identity and the number of sequences in an alignment are two important alignment characteristics. Pair-wise identity is related to the performance of consensus structure folding, while the number of members is important for inferring an accurate evolutionary history. To analyze these two factors, we generated the third benchmarking dataset which consisted of alignments with different number of sequences and identities. Member sequences were randomly picked from each seed alignment. For each family, we generated three sets. Each set included 50 alignments, and the alignments contained 5 sequences, 10 sequences and 20 sequences respectively. 7 families (ctRNA_pGA1, glmS, lin-4, Lysine, mir-10, s2m, Tymo_tRNA-like), whose sequences are less than 50, were excluded from this dataset because the diversity of generated alignments was too small.

PhyloRNAalifold requires a phylogenetic tree of the alignment to infer the consensus structural aspects. In our experiments, DNADIST and KITSCH in the PHYLIP package [45] were used to generate phylogenies. First DNADIST computed a distance matrix of the sequences. After that, KITSCH estimated a phylogenetic tree from the output matrix of DNADIST. The reason of using KITSCH was that it can generate rooted binary trees, which were required by PhyloRNAalifold. Another notable issue in this process is that if two sequences differ in more than 75% of their positions, DNADIST would set the distance between them to -1, which represents infinity. KITSCH rejects negative distances. Thus -1 was replaced with 1000 in distance matrices. We have checked all the positive sequence distances in our experiments. None of them exceeded 10, so 1000 is large enough to represent infinity.

The implementation of PhyloRNAalifold was on the top of program RNAalifold in the Vienna RNA package 2.0.7 [36]. The major change made by PhyloRNAalifold is to incorporate our Fitch module into the scoring scheme of RNAalifold. To test our idea, Matthews correlation coefficient (MCC) [46] was used to measure the accuracy of consensus structure prediction. Its definition is:

(9) $$\;\text{MCC}=\frac{\text{TP}\times \text{TN}-\text{FP}\times \text{FN}}{\sqrt{\left(\text{TP}+\text{FP})(\text{TP}+\text{FN})(\text{TN}+\text{FP})(\text{TN}+\text{FN}\right)}}$$

where *TP*,*TN*,*FP*,*FN* represent the number of true positives, true negatives, false positives, and false negatives, respectively. Additional predicted base-pairs that are not in the reference structure fall into two categories. Some base-pairs contradict reference, the others are compatible with it. Compatible base-pairs can be inserted into the known consensus structure, while adding contradictory base-pairs breaks the pairing rules. Only contradictory base-pairs were counted as false positive predictions.

Five algorithms, RNAalifold, RIBOSUM-based RNAalifold, PhyloRNAalifold, RIBOSUM-based PhyloRNAalifold, and PETfold, have been tested on the first two datasets. The first four have also been benchmarked on the third dataset. The RIBOSUM scoring scheme [47] is used in the latest version of Vienna RNA package. In this scheme, the sum of Hamming distance $h(s_{i}^{k},s_{i}^{l})+h(s_{j}^{k},s_{j}^{l})$ in conservation score was replaced by an entry in the RIBOSUM matrix *R*: $R(s_{i}^{k}s_{j}^{k},s_{i}^{l}s_{j}^{l})$. The experiment results in [34] showed that RIBOSUM-based RNAalifold outperformed the orignal RNAalifold in most of cases. In addition, the authors of [34] used *ϕ*_1_=0.6 and *ϕ*_2_=0.5 as the default parameters in their experiments. We applied their settings to make the comparison fair. The performance of PETfold was tested in our experiments too. We used the web-server of PETfold [48] and its default parameters.

### Effect of parameter ***β***

**Figure 2:**
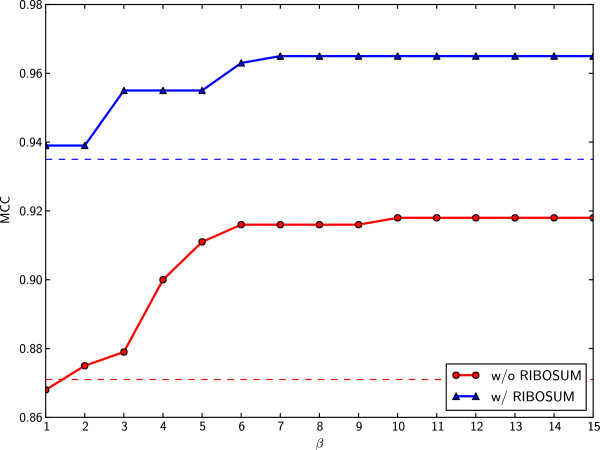
**MCC on the CMfinder dataset as a function of the *β *parameter.** The MCC results of PhyloRNAalifold, with and without RIBOSUM matrix support, are shown in this figure. The dash lines are references for the curves, which show the performance of RNAalifold on the same dataset. It can be seen that except for *β*=1, the new phylogenetic-based covariance scoring scheme improves the performance of RNAalifold.

In the first experiment, we compared the structure prediction results of PhyloRNAalifold with RNAalifold on the original CMfinder dataset. Default values for *ϕ*_1_, *ϕ*_2_ and *γ*_*t*_ were used, and *β* was a variable ranging from 1 to 15. Figure 2 shows that the novel scoring scheme of PhyloRNAalifold improves the performance of RNAalifold in nearly all cases, except *β*=1. The differences of average MCC in both cases, with or without using RIBOSUM matrix, become larger when *β* is increased, and they are maximized at *β*=10. The largest differences are 0.079 and 0.033 for RIBOSUM supported and non-RIBOSUM supported algorithms. After that, the performance of PhyloRNAalifold is not boosted with the increasing of *β*. In the following experiments, we select 10 as a default value for *β*.

### Benchmarking with other methods

Table 1 summarizes all results of the consensus structure predictions on the structural alignments. PhyloRNAalifold with RIBOSUM support achieves the best average MCC score. When RIBOSUM matrix is incorporated, the score difference between PhyloRNAalifold and RNAalifold becomes smaller. This may suggest that by using RIBOSUM matrix, RNAalifold quantifies the conservation among base-pairs more precisely than its original solution. The advantage of using phylogenetic history is swamped by this strategy to some extent. The average specificity scores of five algorithms are the same, while PhyloRNAalifold and PETfold have two largest average sensitivity scores. It is evidence of evolutionary information helping the energy-based folding algorithms to detect more base-pairs with introducing very few errors. An interesting observation is that Cobalamin has relative low MCC scores for RNAalifold. PhyloRNAalifold improves the accuracy of the consensus structure prediction of Cobalamin greatly. In addition, PETfold has the best performance on this family among all five algorithms. We checked the consensus structure of Cobalamin and the predicted results of RNAalifold. One possible reason is that there are too many gaps and non-pairing bases at its pairing columns, which decrease the covariance scores of those columns in RNAalifold greatly. Without the bonus from evolutionary information, those columns cannot be detected by RNAalifold at all.

**Table 1:** **The benchmarking results on the structural alignments of the CMfinder dataset (*β=10)***

**Family**	**#seq**	**MPI**	**RNAalifold**	**PhyloRNAalifold**	**RNAalifold** **with RIBOSUM**	**PhyloRNAalifold** **with RIBOSUM**	**PETFold**
Cobalamin	430	49.7	0.756	0.951	0.591	0.951	**0.976**
ctRNA_pGA1	15	73.0	0.979	0.979	0.979	0.979	**1.000**
Entero_CRE	56	81.7	0.912	0.912	0.916	0.916	**1.000**
Entero_OriR	60	87.4	0.47	0.681	0.703	0.703	**0.747**
glmS	18	57.4	0.973	0.987	**1.000**	**1.000**	0.987
Histone3	52	46.0	**1.000**	**1.000**	**1.000**	**1.000**	**1.000**
Intron_gpII	98	52.3	**1.000**	**1.000**	**1.000**	**1.000**	**1.000**
IRE	62	76.8	0.814	0.854	**0.965**	**0.965**	**0.965**
let-7	67	66.4	0.861	0.967	**1.000**	**1.000**	0.915
lin-4	12	68.8	0.977	**1.000**	**1.000**	**1.000**	0.836
Lysine	47	48.4	0.952	**0.981**	**0.981**	**0.981**	0.952
mir-10	36	67.9	0.789	0.865	**0.957**	**0.957**	0.935
Purine	133	54.7	0.904	**1.000**	**1.000**	**1.000**	0.977
RFN	144	68.1	0.826	0.851	0.826	**1.000**	**1.000**
Rhino_CRE	12	81.4	0.581	0.581	**0.976**	**0.976**	0.750
S_box	433	62.9	0.883	0.924	0.963	**1.000**	0.944
s2m	38	78.3	**1.000**	**1.000**	**1.000**	**1.000**	**1.000**
SECIS	61	41.0	0.972	**1.000**	**1.000**	**1.000**	0.972
Tymo_tRNA-like	28	68.2	0.908	0.908	0.910	0.910	**0.975**
		Mean	0.871	0.918	0.935	**0.965**	0.944
		Specificity	**1.000**	**1.000**	**1.000**	**1.000**	**1.000**
		Sensitivity	0.802	0.881	0.919	**0.952**	0.922

The MCC on structural alignments of the CMfinder dataset is compared among PhyloRNAalifold, RNAalifold and PETfold. The parameter *β* of PhyloRNAalifold is 10. Best performance on the same family is set to **bold**.

Table 2 shows the results on the non-structural alignments of the CMfinder dataset. In this case, RIBOSUM-based PhyloRNAalifold still achieves the highest average MCC score. The performance of RNAalifold with RIBOSUM support is almost the same as that of the top one algorithm. What’s more, PETfold, which has a larger average MCC score in the previous experiment than RIBOSUM-based RNAalifold, falls to third place. This suggests that the evolutionary information at the pairing columns may be disrupted by ClustalW, whose alignment function does not consider secondary structures.

**Table 2:** **The benchmarking results on the non-structural alignments of the CMfinder dataset (*β=10)***

**Family**	**MPI**	**RNAalifold**	**PhyloRNAalifold**	**RNAalifold** **with RIBOSUM**	**PhyloRNAalifold** **with RIBOSUM**	**PETFold**
Cobalamin	43.2	**-0.001**	**-0.001**	-0.002	-0.002	-0.002
ctRNA_pGA1	66.5	0.865	0.889	**0.979**	**0.979**	0.936
Entero_CRE	81.7	0.912	0.912	0.916	0.916	**1.000**
Entero_OriR	87.5	0.694	0.830	**0.965**	**0.965**	0.910
glmS	55.2	0.445	0.566	**0.873**	0.784	0.811
Histone3	45.1	**1.000**	**1.000**	**1.000**	**1.000**	**1.000**
Intron_gpII	46.2	0.760	0.794	**0.826**	**0.826**	0.794
IRE	77.3	0.480	0.480	0.710	0.710	**0.816**
let-7	66.7	0.760	**0.761**	0.666	0.666	0.742
lin-4	64.6	0.523	0.521	0.712	0.712	**0.739**
Lysine	44.0	0.307	0.414	**0.513**	0.484	0.388
mir-10	68.4	0.741	0.820	**0.957**	**0.957**	0.935
Purine	53.8	0.852	**0.977**	**0.977**	**0.977**	0.953
RFN	64.2	0.342	0.309	0.302	0.433	**0.477**
Rhino_CRE	81.4	0.581	0.581	**0.976**	**0.976**	0.750
S_box	56.5	0.430	0.430	0.817	**0.860**	0.750
s2m	78.3	**1.000**	**1.000**	**1.000**	**1.000**	**1.000**
SECIS	36.5	**0.000**	**0.000**	**0.000**	**0.000**	-0.003
Tymo_tRNA-like	64.1	0.691	0.703	**0.768**	**0.768**	0.596
	Mean	0.599	0.631	0.735	**0.737**	0.715
	Specificity	0.947	0.947	0.947	0.947	**0.999**
	Sensitivity	0.486	0.545	0.689	**0.704**	0.655

The MCC on non-structural alignments of the CMfinder dataset is compared between PhyloRNAalifold, RNAalifold and PETfold. The parameter *β* of PhyloRNAalifold is 10. Best performance on the same family is set to **bold**.

### Effects of identity and the number of sequences

**Figure 3:**
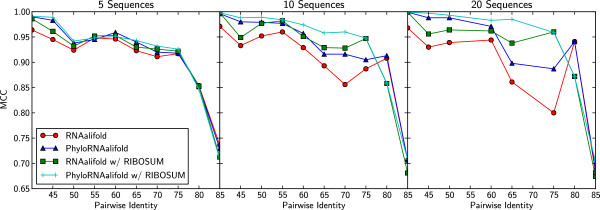
**The effect of alignment pair-wise identity and sequence number on the structural prediction of PhyloRNAalifold (*β=10*).** The MCC results of PhyloRNAalifold and RNAalifold on the third benchmarking dataset are shown in this figure. It can be seen that the performance difference between PhyloRNAalifold and RNAalifold increases with the increasing of the sequence number in the alignments. In addition, the maximum MCC difference is achieved in the range of 65∼75 identities.

In this experiment, we try to analyze the correlation of two alignment characteristics, pair-wise identity and the number of sequences, with the performance of PhyloRNAalifold. Figure 3 shows the experiment results. It can be seen that all four algorithms have similar performance when the number of sequences in the alignments is 5. With the increasing of the members in the alignments, the average MCC difference between PhyloRNAalifold and RNAalifold becomes larger. Using more sequences provides a more precise phylogenetic history, so it is reasonable that PhyloRNAalifold achieves its best performance on alignments with 20 sequences. In addition, for experiments on the alignments of 10 sequences and 20 sequences, the maximum performance difference exists between the pair-wise identities 65 and 80. If the pair-wise identity of an alignment is small, the original covariance scoring scheme of RNAalifold works well enough because a large number of different base-pairs at the pairing columns provide substantial conservational signals. On the other hand, when the alignment’s pair-wise identity is too large, all the symbols at the pairing columns are almost the same. The effect of our new covariance scoring scheme is reduced due to the lack of evolutionary information.

## Discussion and conclusion

We have proposed a novel approach, PhyloRNAalifold, to fold RNA consensus structures by evaluating the level of conservation in aligned RNA sequences. With an evolution-based covariance scoring scheme, PhyloRNAalifold can detect more potential pairing columns than RNAalifold. The benchmark testing shows that PhyloRNAalifold can improve the performance of RNAalifold, as well as PETfold.

There are two possible directions for further research. The first direction is to analyze the dependence of PhyloRNAalifold on the phylogenetic tree construction algorithms. Tree structures have great effect on the RNA structure prediction of PhyloRNAalifold. Besides DNADIST and KITSCH, there are other algorithms, such as UPGMA [49], PAUP [50] and MrBayes [51], which can construct alternative trees. Finding or design an optimal algorithm for detecting the phylogenetic information in the pairing columns is an open question. Ideally, structure conservation should be considered because it is crucial for evaluating the similarity between two RNA sequences. The second direction deals with incorporating the phylogenetic information of non-pairing bases into the folding algorithm. Only covarying mutations among base-pairs are considered in PhyloRNAalifold. In probabilistic methods, all the possible mutations, including mutations in loop regions and stack regions, are modeled with HMM. Our goal is to incorporate both types of mutations into PhyloRNAalifold, while still keep the simplicity of the scoring scheme.

## Availability

The latest version of PhyloRNAalifold can be downloaded at: http://genome.ucf.edu/PhyloRNAalifold.

## Competing interests

Both authors declare that they have no competing interests.

## Authors’ contributions

SZ conceived of the study and designed the algorithm. PG implemented the algorithm and carried out the benchmarking tests. Both authors wrote and approved the manuscript.
